# Trial protocol for a randomised controlled trial of red cell washing for the attenuation of transfusion-associated organ injury in cardiac surgery: the REDWASH trial

**DOI:** 10.1136/openhrt-2015-000344

**Published:** 2016-03-07

**Authors:** G J Murphy, V Verheyden, M Wozniak, N Sullo, W Dott, S Bhudia, N Bittar, T Morris, A Ring, A Tebbatt, T Kumar

**Affiliations:** 1 Department of Cardiovascular Sciences and NIHR Cardiovascular Biomedical Research Unit, University of Leicester, Clinical Sciences Wing, Glenfield Hospital, Leicester, UK; 2 University Hospitals Coventry and Warwickshire NHS Trust, Coventry, UK; 3 Blackpool Victoria Hospital NHS Trust, Blackpool, UK; 4 Leicester Clinical Trials Unit, Leicester Diabetes Centre, Leicester General Hospital, Leicester, UK; 5 Department of Clinical Perfusion, University Hospital Leicester NHS Trust, Glenfield Hospital, Leicester, UK

**Keywords:** CARDIAC SURGERY

## Abstract

**Introduction:**

It has been suggested that removal of proinflammatory substances that accumulate in stored donor red cells by mechanical cell washing may attenuate inflammation and organ injury in transfused cardiac surgery patients. This trial will test the hypotheses that the severity of the postoperative inflammatory response will be less and postoperative recovery faster if patients undergoing cardiac surgery receive washed red cells compared with standard care (unwashed red cells).

**Methods and analysis:**

Adult (≥16 years) cardiac surgery patients identified at being at increased risk for receiving large volume red cell transfusions at 1 of 3 UK cardiac centres will be randomly allocated in a 1:1 ratio to either red cell washing or standard care. The primary outcome is serum interleukin-8 measured at 5 postsurgery time points up to 96 h. Secondary outcomes will include measures of inflammation, organ injury and volumes of blood transfused and cost-effectiveness. Allocation concealment, internet-based randomisation stratified by operation type and recruiting centre, and blinding of outcome assessors will reduce the risk of bias. The trial will test the superiority of red cell washing versus standard care. A sample size of 170 patients was chosen in order to detect a small-to-moderate target difference, with 80% power and 5% significance (2-tailed).

**Ethics and dissemination:**

The trial protocol was approved by a UK ethics committee (reference 12/EM/0475). The trial findings will be disseminated in scientific journals and meetings.

**Trial registration number:**

ISRCTN 27076315.

**Key questions**

**What is already known about this subject?**

- Changes in allogenic red blood cells and the accumulation of toxic intermediaries in donor blood bags during storage are thought to contribute to the development of organ injury in transfusion recipients.
- Studies in animal models have suggested that removing the storage supernatant from allogenic red cells by mechanical cell washing may attenuate the inflammation and organ injury associated with transfusion.

**What does this study add?**

- The objective of the REDWASH study is to determine whether mechanical washing of donor red cells will reduce inflammation and organ injury in adult cardiac surgery patients that receive large volume red cell transfusions.

**How might this impact on clinical practice?**

- Mechanical red cell washing devices are widely used in cardiac surgery for the processing of shed autologous blood. If effective, this intervention could we widely adopted.

## Introduction

### The clinical problem

Cardiopulmonary bypass and other surgical techniques utilised in cardiac surgery are associated with a high incidence of organ injury and dysfunction that typically affects the kidney, heart and lungs.^1^ Organ injury affects up to 30% of all adult cardiac surgery patients and is associated with additional morbidity, high mortality and the increased use of healthcare resources.^2–5^ The underlying pathophysiological processes are poorly understood; there are no current interventions that have been shown to alter the natural history of these conditions and outcomes remain poor.^6–9^ As the population ages and increasingly elderly patients with more comorbidity are referred for cardiac surgery,^10^ it is likely that the risk of inflammatory organ injury will increase. Refining perioperative care to reduce the frequency and severity of inflammatory organ injury is therefore a clinical research priority.

### Blood transfusion and adverse outcome in cardiac surgery

Red cell transfusion is the preferred treatment for acute blood loss or the rapid reversal of anaemia in cardiac surgery patients. These indications are common, and red cell transfusion is also common, with over half of all cardiac surgery patients receiving red cells on average.^11^ ^12^ However, red cell transfusion is strongly associated with adverse clinical outcomes including organ injury, infection and death.^13^ ^14^ The clinical importance of these associations is uncertain. A recent meta-analysis of randomised trials has indicated that allogenic red cells may be beneficial in cardiac patients with severe anaemia.^15^ However, the volumes of red cells transfused for anaemia in these trial were small, typically 1–2 units, and there is uncertainty as to whether larger volumes of red cells, more commonly associated with severe bleeding, may have a different balance of risks and benefits. This is suggested by the much higher risk of mortality observed with large versus small volume transfusions in observational analyses (OR for death 1.73 and 2.68 for 1–2 units and 3–5, respectively^15^), and the much greater risk of death and organ injury in cardiac surgery patients that have been treated for severe blood loss.^16^ Observational studies cannot demonstrate causality however, and it is also not possible to randomise critically ill patients, many of whom are bleeding to red cell transfusion or no red cell transfusion. One approach to address this uncertainty is to modify red cells, to assess whether attenuation of the adverse effects of storage results in a reduction in adverse clinical outcomes. Intuitively, the benefits of these interventions would be greatest in recipients of large volume blood transfusion (LVBT). LVBT, defined as 4 or more units or greater than 1000 mL transfused^17^ is common, affecting up to 22% of patients in a 2010 audit of UK centres 22%, ranging from 8% to 45% between units.^11^ We have previously developed a preoperative risk score that can identify patients at risk of receiving LVBT with excellent discrimination.^17^ We will use this tool to identify an enriched cohort of patients that we believe may benefit most from our proposed blood safety intervention.

### The pathogenesis of transfusion-mediated organ injury

**Figure 1:**
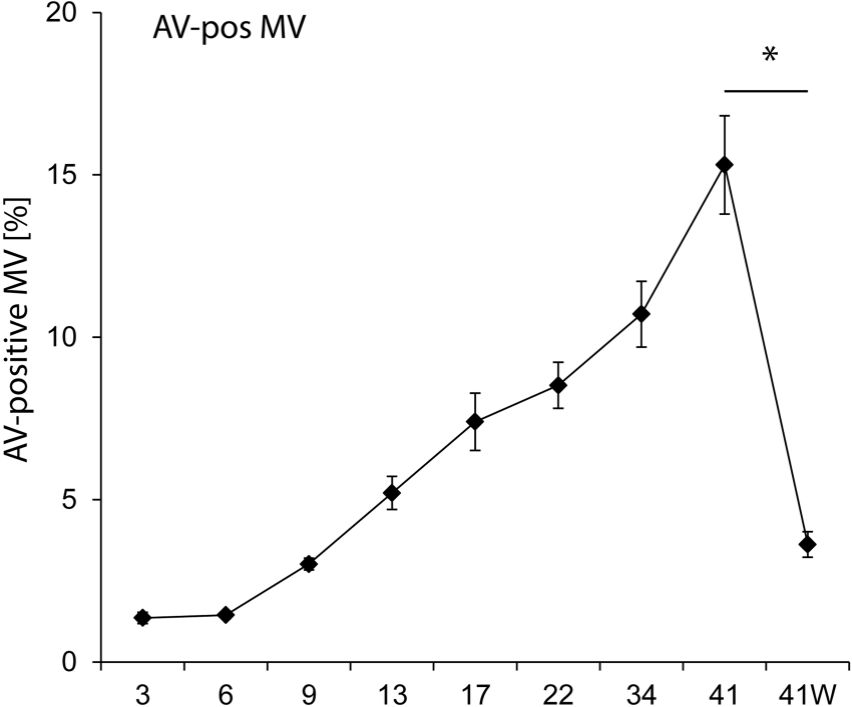
Annexin V (AV) positive microvesicles (MV) in allogenic red cell stored in SAGM at 4°C for up to 42 days and after washing with a Fresenius Continuous AutoTransfusion System (CATS, Fresenius AG, Bad Homburg, Germany). SAGM, saline adenine glucose mannitol.

Organ injury and immunomodulation following transfusion have been attributed to the ‘storage lesion’; changes in red cell properties and accumulation of inflammatory particles in the supernatant of red cell units during storage.^18^ In experimental studies, we have shown that these changes cause inflammatory organ injury via complex mechanisms including platelet and monocyte activation, endothelial injury and oxidative stress and the loss of microcirculatory autoregulation.^19–22^ This results in paradoxical tissue hypoxia despite apparently adequate oxygen delivery, tissue inflammation, and organ dysfunction. The most important change that occurs in stored red cells appears to be the depletion of high energy phosphates in red blood cell (RBC) over time.^18^ This leads to the loss of autoregulatory function, erythrocyte deformability and changes in erythrocyte morphology that are associated with abnormal gas transfer and microcirculatory flow. A significant aspect of these changes is the overexpression of phosphatidylserine (PS) on the RBC surface attributable to diminished function of the ATP-dependent membrane-bound flippase enzyme that acts to maintain membrane asymmetry by transporting PS to the interior of the membrane and phosphatidylcholine to the exterior.^23^ High levels of PS expression are associated with rapid uptake of donor RBC by recipient myeloid tissue and hence diminished donor cell survival. They are also associated with the release of membrane vesicles from the erythrocytes during storage^24^ (figure 1).

These microvesicles (MV) express high PS levels that have potent inflammatory effects.^25^ Our own research has indicated that MV levels increase progressively from the onset of storage, and that they may be removed by mechanical cell washing (figure 1). Furthermore, in a porcine model, we have shown that mechanical washing of allogenic RBC to remove these microparticles reduces inflammation and inflammatory organ injury in transfusion recipients (unpublished). In this study, washing cells that exhibited storage-related changes typical of human cells reversed platelet and endothelial activation and pulmonary injury in swine. On the basis of these results, we now hypothesise that the release of microparticles by erythrocytes during storage is associated with inflammatory organ injury in cardiac surgery patients receiving blood transfusion and that this may be prevented by red cell washing prior to transfusion. We propose to test this hypothesis in the current trial.

### Red cell washing

Red cell washing devices are ubiquitous in cardiac surgery. The salvage, washing, transfusion of *autologous* blood lost from the operative field is part of standard care, and has been shown to improve clinical outcomes, perhaps by reducing allogenic RBC exposure.^26^ Washing of *allogenic* RBC is also practiced in paediatric cardiac surgery. Low birthweight neonates and small infants are susceptible to the high ion (free haemoglobin, potassium, calcium) concentrations that are present in older RBC units and it is the practice in some centres to wash these using cell salvage devices prior to transfusion.^27^ A randomised controlled trial (RCT) of red cell washing in paediatric cardiac surgery patients failed to show a clinical benefit, although there was a reduction in measures of inflammation (interleukin (IL)-6:IL-10 ratios). Importantly, no adverse effects of this technique were reported in recipients.^28^

Standard cell saver devices use low speed centrifugation with resuspension in normalised saline without apparent detriment to human autologous or allogenic RBC.^29^ ^30^ Our own studies (unpublished) have also documented significant homology between the effect of mechanical red cell washing using low speed centrifugation with the Fresenius Continuous AutoTransfusion System (CATS, Fresenius AG, Bad Homburg, Germany) and the washed red cells produced by the UK National Blood Service for patients at increased risk of hypersensitivity reactions. We suggest that the pretransfusion washing of stored donor cells using commonly used cell salvage devices at the bedside will offer a simple and practical blood safety intervention.

## Aims and objectives

The REDWASH trial will test the hypothesis that the severity of the postoperative inflammatory response will be less and postoperative recovery faster if patients undergoing cardiac surgery with cardiopulmonary bypass (CPB) who are at risk of large volume RBC transfusion receive stored allogenic RBC that are washed prior to transfusion when compared with standard care where stored RBC are administered without washing. A secondary hypothesis is that the adverse effects of transfusion are mediated by platelet and monocyte activation by microparticles within the storage supernatant and that by removing the supernatant this is attenuated.

Specific objectives of this trial are to:

- Estimate mean differences in biochemical markers of the systemic inflammatory response between participants allocated to receive washed versus unwashed RBC.
- Estimate mean differences in hospital length of stay between participants allocated to receive washed versus unwashed RBC.
- Estimate differences in the frequency of inflammatory organ injury or death between participants allocated to receive washed versus unwashed RBC.
- Estimate the cost-effectiveness of washed versus unwashed RBC.
- Establish whether red cell washing attenuates postoperative platelet, endothelial cell and monocyte activation (mechanism substudy).

## Methods and analysis

### Study design

This study is a multicentre, single-blinded, parallel group RCT of washing of allogenic RBC prior to transfusion versus standard care (no washing).

### Study population and recruitment

The study will be carried out at three tertiary cardiac surgery centres in the UK: the University Hospitals of Leicester National Health Service (NHS) Trust, the Royal Victoria Hospital, Blackpool and University Hospitals Coventry and Warwickshire NHS Trust. These units perform over 3500 major cardiac procedures per year, of whom over 875 will be at risk of LVBT (from the UK National Audit of Blood Transfusion in Cardiac Surgery^11^). If red cell washing is effective, its clinical benefits and impact on resource use will be most apparent in patients at greatest risk of LVBTs. Patients at risk of LVBT will therefore be identified preoperatively using a risk score developed and validated by these investigators in a multicentre population.^17^

#### Inclusion criteria

Participant may enter study if ALL of the following apply:

1. Adult cardiac surgery patients (≥16 years) undergoing cardiac surgery with blood cardioplegia.
2. Identified as representing a high-risk group for LVBT using a modified risk score. The score for inclusion ≥25. This has 55% positive predictive value for LVBT.^17^

#### Exclusion criteria

Participant may not enter study if ANY of the following apply:

1. Emergency or salvage procedure.
2. Ejection fraction <20%, that is, very poor left ventricular function.
3. Patients with end stage renal failure defined as an estimated glomerular filtration rate (eGFR) <15 mL/min/1.72m^2^ calculated from the Modification of Diet in Renal Disease equation,^31^ or patients who are on long-term haemodialysis or have undergone renal transplantation.
4. Patients who are prevented from having blood and blood products according to a system of beliefs (eg, Jehovah's Witnesses).
5. Patients with congenital or acquired RBC, platelet or clotting factor disorders (excluding those receiving antiplatelet therapy, warfarin or other systemic oral anticoagulants).
6. Patient in a critical preoperative state (Kidney Disease: Improving Global Outcomes (KDIGO) stage 3 acute kidney injury (AKI)^32^ or requiring inotropes, ventilation or intra-aortic balloon pump) preoperatively.
7. Pregnancy.
8. Patients who are participating in another interventional clinical study.

### Intervention being investigated

#### Treatment regimens

Patients will be screened by the investigators to assess eligibility for entry into the study. Eligible patients undergoing cardiac surgery with CPB who consent to participate will be randomly allocated, in a 1:1 ratio to:

1. Group A: unwashed RBC (standard care);
2. Group B: washed RBC.

#### Red cells

Allogenic RBC, harvested in citrate-adenine-phosphate-dextrose, buffy coat removed, leucocyte depleted, saline-adenine-glucose-mannitol stored red cell units, supplied by National Health Service Blood and Transplant as per standard practice will be used. For the intervention, each unit of RBC will be added to a continuous autotransfusion system (CATS, Fresenius AG, Bad Homburg, Germany), washed using a centrifugal method, as per the device instructions. The washed RBC will then be immediately administered to the patient as per standard practice. Washing may be impractical in bleeding patients where there is cardiovascular instability, and clinician discretion may lead to the administration of unwashed cells in the washed group in breach of protocol. The clinical indication and timing of every transfusion, and whether or not washing has occurred as indicated will be recorded. The haematocrit (Hct) threshold for transfusion will be 23. Clinician discretion may also allow variation in this threshold in certain situations, that is, bleeding, where Hct thresholds are impractical.

#### Administration of study treatment

RBC units (washed or unwashed) will be administered via the appropriate giving set, preferably 1 unit at a time. Before transfusion of washed or unwashed cells, the anaesthetist or intensive care unit (ICU) staff will check to ensure that the blood bag has the correct participant identification as per standard care. Because the washed RBC contains no additive solution, the washed RBC must be administered as soon as possible after its preparation. Cell washing and administration in the theatre and ICU will adopt policies as for the washing and transfusion of autologous blood; that is, after appropriate identification checks, the cells will be washed at the patient's bedside and administered immediately. Once washed, RBC units will not be stored for future use.

#### Deviations from protocol

In the event of any deviation from the trial protocol, defined as the administration of a washed unit to a patient randomised to the unwashed arm or vice versa, the deviation will be documented and the patient will continue to be treated according to the randomised allocation for all subsequent transfusions.

### Primary and secondary end points

#### Primary outcome

**Figure 2:**
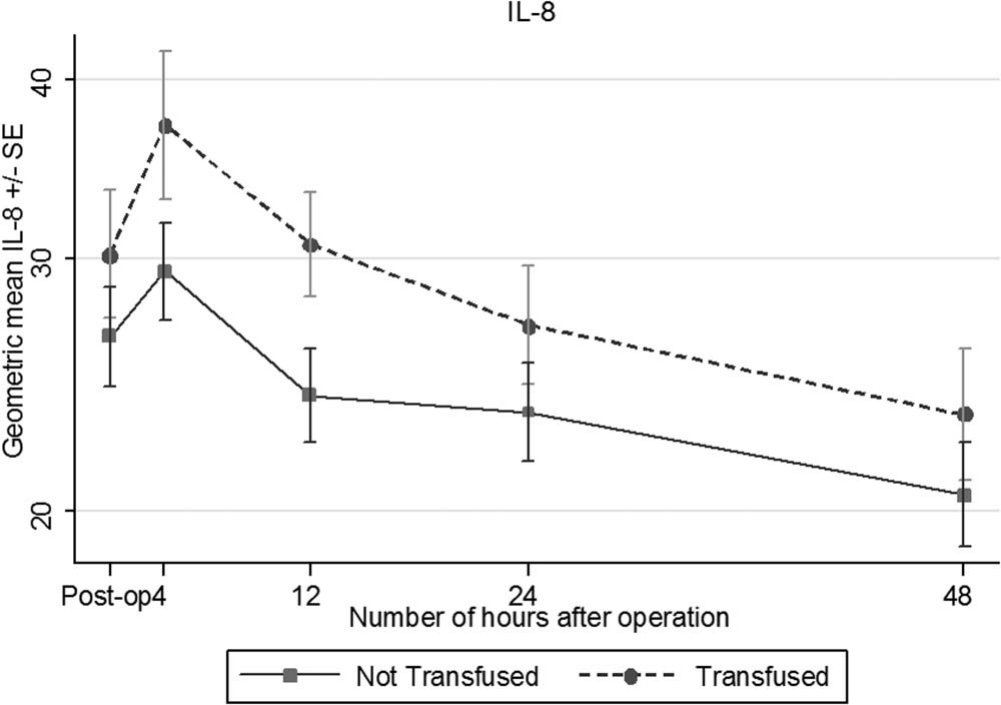
Individual patient data meta-analysis showing serial interleukin (IL)-8 levels measured up to 48 h postsurgery in transfused and non-transfused patients from historical data.

The primary outcome is serum IL-8 levels measured from venous blood samples taken at five postoperative time points; on return to intensive therapy unit (ITU), and 6, 24, 48 and 96 h, adjusted for baseline (preoperative) values. IL-8 levels reflect the severity of the systemic inflammatory response and are an important predictor of adverse clinical events in cardiac surgery patients.^33^ ^34^ Our previous research^35^ has indicated that blood transfusion is associated with increases in the levels of this proinflammatory cytokine that are maximal between 4 and 24 h postoperatively (figure 2). However, the intervention period in the REDWASH trial extends to 48 h postsurgery and therefore IL-8 levels will be measured up to 96 h to capture events after this point.

#### Secondary outcome

The secondary outcome measures are listed in table 1.

**Table 1:** Secondary outcomes

Outcome	Definition/method of verification
Inflammatory organ injury, sepsis or death	▸ Sepsis will be defined as antibiotic treatment for suspected infection, *and th*e presence of SIRS within 24 h prior to start of antibiotic treatment where SIRS is defined as ≥2 of the following conditions: temperature >38^o^C or <36^o^C; heart rate >90 bpm; respiratory rate >20 breaths/min or PaCO_2_ <32 mm Hg; white cell count >12 000/mm^3^ or <4000/mm^3^, *or* antibiotic treatment for wound infection.^36^ ▸ Acute kidney injury, defined as KDIGO^32^ stage 1, 2 or 3. ▸ Acute lung injury, defined as PaO_2_/FiO_2_ ratio <300 mm Hg or a requirement for respiratory support; invasive ventilation>48 h, non-invasive ventilation>4 h, reintubation, tracheostomy, or ARDS.^37^ ▸ Low cardiac output, defined as new intraoperative or postoperative intra-aortic balloon pump insertion OR a cardiac index of <2.2 L/min/m^2^ measured using a Swann Ganz catheter that is refractory to appropriate intravascular volume expansion after correction or attempted correction of any dysrhythmias, OR the administration of inotropes including dobutamine, enoximone, milrinone, levosimendan and adrenaline. ▸ Death. ▸ Differences in Multiple Organ Dysfunction Score^38^ at days 1, 2, 3 and 5.
Bleeding and transfusion	▸ Measured blood loss in drains at 6 h postoperatively. ▸ The number of units of RBC and other blood components transfused during the operative period and postoperative hospital stay will be recorded. ▸ Age of each unit of RBC transfused. ▸ Serial haemoglobin levels/haematocrit.
Transfusion reactions	▸ Febrile transfusion reactions. ▸ Non-haemolytic transfusion reactions. ▸ Haemolytic transfusion reactions. ▸ As defined in http://www.shotuk.org/wp-content/uploads/SHOT-Definitions-Jan-2015.pdf
Other clinical outcomes	▸ Stroke; diagnosed by brain imaging (CT or MRI), in association with new onset focal or generalised neurological deficit (defined as deficit in motor, sensory or coordination functions). ▸ ST elevation myocardial infarction accompanied by troponin I >5000 pg/mL.
Hospital stay, cumulative resource use and quality of life	ICU, HDU and hospital length of stay will be determined by the assessment of care level. Resource use will be costed using credible nationally published sources. Postdischarge resource assessed using a Health Resource Use Questionnaire at 6 weeks and 3 months postsurgery. Quality-adjusted life years assessed using the EuroQol EQ-5D^39^ questionnaire at baseline and at 6 weeks and 3 months postsurgery.
Compliance with the washing protocol	Data will be collected for all patients during surgery to characterise compliance with the randomly assigned washing protocol.
Additional markers of inflammation and organ injury will be assessed in a mechanism substudy in the first 60 consecutive patients recruited at Glenfield Hospital	▸ Urinary LFABP, NGAL at baseline and at 6, 12 and 24 h.^40^ ^41^ ▸ Serum troponin I at baseline and at 24 and 48 h. ▸ Platelet aggregation (Multiplate) in the first 48 h. ▸ Transfused RBC characteristics (washed and unwashed); ATP levels, 2,3DPG, deformability, osmotic fragility, cytokine levels. ▸ Serum levels of GM-CSF, IFN-γ, IL-1β, IL-2, IL-4, IL-5, IL-6, IL-10 and TNF-α at the same time points as for the primary end point. ▸ Platelet and monocyte activation as determined by flow cytometry for a subgroup of patients. ▸ Endothelial injury as determined by quantification of endothelial-derived microparticles by flow cytometry. ▸ Effect of blood harvested from recipients on platelet and monocyte activation within a microfluidics system.

ARDS, adult respiratory distress syndrome; FiO_2_, fractional inspired oxygen; GM-CSF, granulocyte-macrophage colony-stimulating factor; HDU, high dependency unit; ICU, intensive care unit; IFN, interferon; KDIGO, Kidney Disease: Improving Global Outcomes; LFABP, liver fatty acid binding protein; NGAL, neutrophil gelatinase-associated lipocalin; PaCO_2_, arterial carbon dioxide tension; PaO_2_, arterial oxygen tension; RBC, red blood cell; SIRS, systemic inflammatory response syndrome; TNF, tumour necrosis actor.

### Duration of treatment in the trial

The intervention is transfusion of any allogenic blood product between the start of surgery and 48 h postoperatively.

### End of the trial

For an individual participant, the end of the trial is defined as completion of the 3-month postal follow-up assessment.

### Clinical management of study participants

#### Administration of non-RBC blood components

Patients receiving LVBT frequently receive non-RBC components. These will be administered according to standard unit protocols, with the indication, volume and timing of their administration recorded. Platelet transfusions will not be washed in this study.

#### Blood management adjuncts

A single cell salvage device will be used for allogenic and autologous red cell washing and transfusion. Intraoperative cell salvage will be used in every patient with washing, resuspension in normal saline and autotransfusion as per standard care. Cardiotomy suction will be returned directly to the extracorporeal circuity without washing. Postoperative salvage of mediastinal fluid will not be performed. Tranexamic acid will be administered to every patient as per the BART (Blood Conservation Using Antifibrinolytics in a Randomized Trial) protocol.^42^ In patients refractory to two standard doses of non-RBC blood components (a standard dose=1 pooled adult platelets and 2–4 units of fresh frozen plasma, 2 units of cryoprecipitate), recombinant activated factor VII or prothrombin complex concentrate, may be administered at the discretion of the attending clinician.

#### Concomitant treatment

Patients may receive medications and/or other therapies to treat adverse events as deemed necessary by the investigator or the patient's physician. Concomitant medications and/or therapy that become necessary during the study and any changes in concomitant medication and/or therapy will be recorded on the case report forms (CRFs). Details of concomitant medications and therapy will include generic drug name, dose, route, duration and indication.

#### Preoperative care

Eligible patients will receive standard care preoperatively.

#### Anaesthesia

A standard anaesthetic protocol will be used. Patients will undergo anaesthetic induction with midazolam/propofol/fentanyl (up to15 µg/kg) and short-acting muscle relaxant. Anaesthetic maintenance will use isoflurane/sevoflurane until start of cardiopulmonary bypass where propofol (1%, Diprivan, 20–40 mL/h) maintenance will be started until the end of the procedure. Target perfusion pressures (mean 70–80) will be maintained initially with incremental metaraminol or phenylephrine boluses (0.5 mg) or vasodilators, and post bypass with inotropic support as necessary. Morphine 10–15 mg and paracetamol 1 g are administered intravenously on chest closure to facilitate postoperative analgesia. Postoperative analgesia comprises regular paracetamol and morphine patient controlled analgesia (PCA) for up to 48 h where regular oral tramadol 50–100 mg is started. The anaesthetic agents will be recorded in the patient CRF.

#### Cardiopulmonary bypass

Cardiopulmonary bypass will be managed according to a standard CPB protocol. Normothermic to mild hypothermic non-pulsatile or pulsatile CPB (32–35°C) will be established using a standard venous reservoir, a roller pump, a hollow fibre oxygenator and a non-heparin-bonded circuit with target flows of 2.4–2.7 L/min/m^2^, and mean arterial blood pressure (MABP) maintained between 60 and 80 mm Hg. Circuit prime will typically include 1000 mL ringers lactate, 500 mL gelofusin, mannitol 20% and 5000 IU heparin. Intermittent antegrade/retrograde blood cardioplegic arrest will be performed. Hct will be maintained >23. Target-activated clotting time of >400 s will be achieved with heparin (300 µ/kg as a loading dose) for bypass. Heparin reversal will be achieved with the administration of protamine sulfate in a 1:1 ratio as per standard practice.

#### Postoperative care

Intravenous glycopyrolate, atropine, atrial or dual chamber epicardial pacing will be used to achieve a target heart rate (70–110 bpm). The use of inotropes or vasopressors will be at the discretion of the attending physician. Postoperative oliguria, defined as a urine output <0.5 mL/kg/h for four consecutive hours will be treated initially with fluid boluses to maintain the central filling pressure >12 mm Hg, and then inotropes (enoximone, dobutamine, epinephrine) or pressor agents (norepinephrine or vasopressin) as indicated to maintain adequate perfusion pressure (ie, MABP >80 mm Hg or within 10% of preoperative MABP), or cardiac output (ie, cardiac index >2.1 L/min/m^2^) as determined by appropriate invasive monitoring. Persistent oliguria resistant to these measures may be managed by forced diuresis, using, for example, furosemide. Decisions about discharge from ICU, high dependency unit (HDU) and from hospital will be made on the basis of existing institutional protocols.

### Research procedures

#### Screening and eligibility assessment

The patients risk score will be calculated at the preoperative assessment clinic or from our standard in patient referral protocols that include detailed clinical and demographic information (table 2). An information leaflet, approved by the local Research Ethics Committee, will be sent to all potentially eligible patients waiting at home with the letter giving a date for their operations. Each patient will have at least 24 h to consider whether to participate or not. In a few cases, this time interval may be as little as 12 h, for example, for patients admitted for urgent surgery without prior notification to the waiting list coordinator. Despite the short notice, it is important to include these patients for the applicability of the trial findings since about 40% of patients having cardiac surgery are admitted as urgent cases, and these are often those at greatest risk for LVBT. Written informed consent will be obtained at the time of admission. Details of all patients approached for the trial and reason(s) for non-participation (eg, reason for being ineligible or patient refusal) will be documented.

**Table 2:** Key data collection points

	Preoperation	Operation day	Day 1	Day 2	Day 3	Day 4	Discharge	6 weeks	3 months
Eligibility	✓								
Written consent	✓								
Randomisation	✓								
EQ5D Questionnaire	✓						✓	✓	✓*
Bloods: serum biochemistry (creatinine and troponin T/I)	✓†	✓† (CICU and 6–12 h)	✓† (24 h)	✓† (48 h)	✓† (72 h)	✓‡ (96 h)	✓‡		
Bloods: serum inflammatory biomarkers	✓†	✓† (CICU and 6–12 h)	✓† (24 h)	✓† (48 h)	✓† (72 h)	✓‡ (96 h)	✓‡		
Bloods: full blood counts	✓	✓ (CICU and 6–12 h)	✓ (24 h)	✓ (48 h)	✓ (72 h)	✓‡ (96 h)	✓‡		
Bloods: plasma sample for MP analysis and monocyte activation	✓	✓ (CICU)	✓ (24 h)	✓ (48 h)					
Urine sample and volume: NGAL, urea and elecrolytes	✓	✓ (6 and 12 h)	✓ (24 h)	✓†§ (48 h)					
Operative details		✓†							
Clinical outcomes						✓	✓	✓¶	
Serious adverse event monitoring		✓	✓	✓	✓	✓	✓	✓¶	
Resource use data							✓	✓¶	✓*
Bloods: platelet response**	✓**	✓** (CICU and 6–12 h)	✓** (24 h)	✓** (48 h)					

*Indicates data collection via postal questionnaire.

†Indicates samples taken as part of normal care.

‡Discharge time point if hospital stay exceeds 5 days.

§Indicates sample for determination of routine urea and electrolytes only.

¶The 4–6-week time point in accordance with normal postoperative care.

**Indicates Glenfield patients alone.

CICU, cardiac Intensive care unit; NGAL, neutrophil gelatinase-associated lipocalin.

#### Randomisation and code breaking

Patients will be randomly assigned in a 1:1 ratio using an internet-based randomisation system (Sealed Envelope Ltd, MHRA recognised facility). Randomisation will be stratified by: (1) study site; (2) type of procedure, coronary artery bypass grafts (CABG), valve, CABG and valve, other. Random allocations will be generated only after the relevant baseline data to identify the patient and the surgeon have been entered into the system, guaranteeing concealment of allocation and a definitive log of participants. Patients who consent will be randomised by a member of the research team at a participating site. If patients are unexpectedly rescheduled, they will retain their study numbers and randomised allocation.

**Figure 3:**
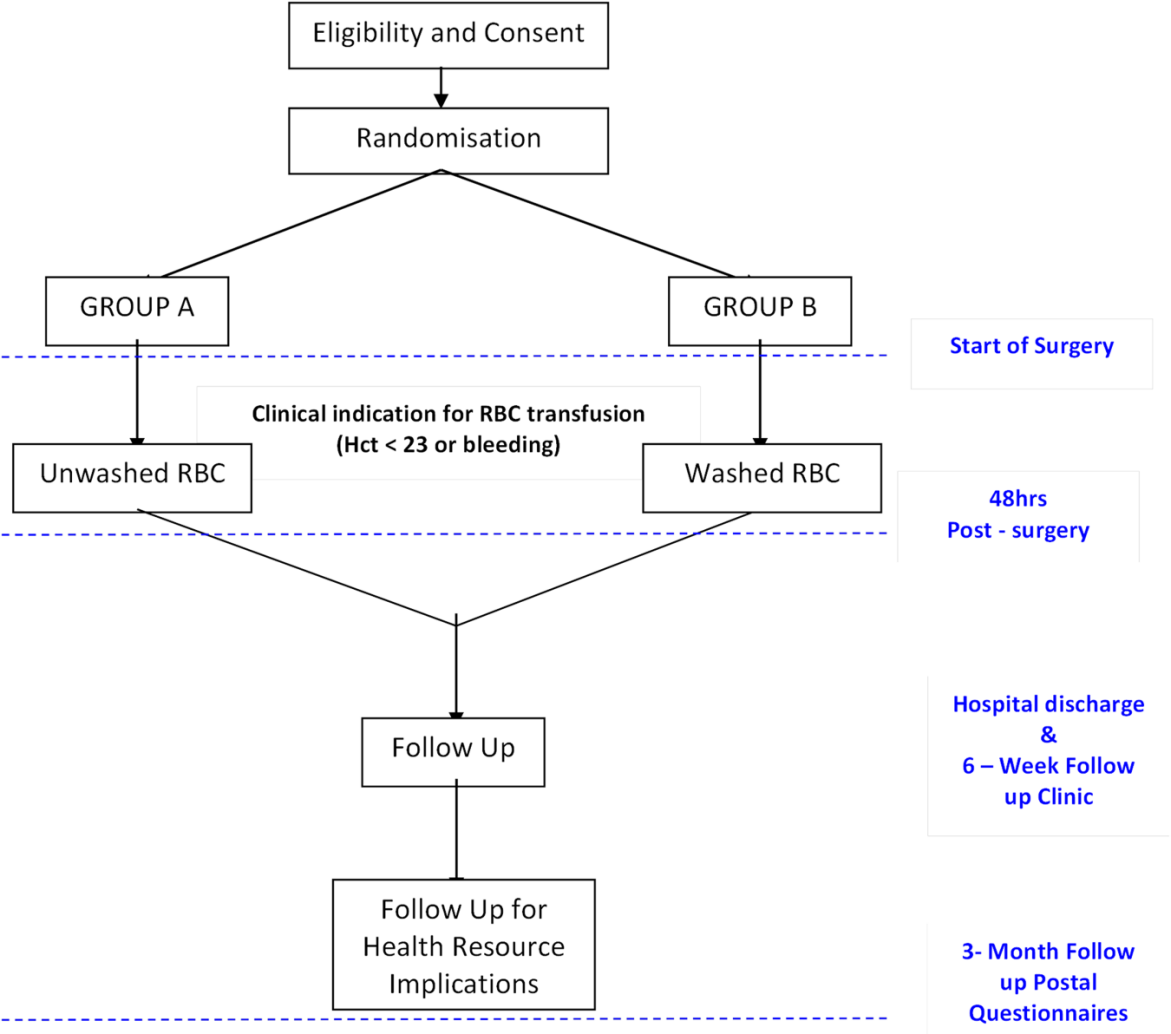
Patient flows showing randomisation, intervention period and follow-up period. Hct, haematocrit; RBC, red blood cell.

Detailed instructions for the randomisation process will be provided in a separate manual (figure 3).

#### Trial-specific tests and procedures

Participants will undergo the following tests and procedures as part of the research.

*Urine samples* will be collected on the day before surgery, on the day of surgery (6 and 12 h postsurgery) and day 1 (24 h postsurgery) for the measurement of AKI biomarkers.

*Blood samples* will be collected from all participants in the trial at the following time points: preoperatively, postoperatively, on return to ITU, 6–12 h postoperative, 24 h postoperative, 48 h postoperative, 72 h postoperative, 96 h postoperative or hospital discharge, whichever is the earliest (ie, seven blood samples will be taken per patient). Inflammatory markers in serum; granulocyte-macrophage colony-stimulating factor (GM-CSF), interferon-γ, IL-1β, IL-2, IL-4, IL-5, IL-6, IL-8, IL-10 and tumour necrosis factor-α will be measured on the MagPix Multiplex platform (Luminex Corp). Preoperatively, on return to cardiac Intensive care unit (CICU) and 24 and 48 h postoperative, additional blood will be collected for analysis of MP levels and platelet and monocyte activation using flow cytometry (Beckman Coulter MCL-XK, High Wycombe, UK) and multiplate impedance electrode aggregometry (Roche, Rotkreuz, Switzerland).

Urine and blood samples will be transferred to the central laboratory where they will be stored until analysed for cytokines, coagulopathy, inflammatory cell activation, endothelial injury and inflammatory organ injury biomarkers. The samples will be kept until the completion of the study. Left over material may be used for future research with permission of the research participants. Full blood count analysis will be conducted at the local haematology laboratories of the participating sites.

*Assessment of cumulative resource use and utility* will be carried out for all participants: Utility will be assessed using the EuroQol EQ5D^39^ assessed at baseline, 6 weeks (routine clinic visit) and 3 months after surgery (postal questionnaire). Hospital resource use will be directly measured using available reference costs.^43^ Postdischarge resource use will be assessed using a health resource usage questionnaire conducted at 6 weeks (routine clinic visit) and 3 months after surgery (postal questionnaire) as previously described.^36^

#### Planned recruitment rate

On the basis of historical data, we estimate that there will be a target population of about 1300 (<25%) eligible from 5250 total patients over 18 months at all three centres (from the UK National Transfusion Audit and 2011–2012 activity.^11^ We plan to recruit 170, or 13% of all eligible patients lower than that regularly achieved in other trials carried out by these researchers.

#### Discontinuation/withdrawal of participants

Each participant has the right to withdraw at any time. It is unlikely for this trial that there would be any reason for the investigator to withdraw any participant, or for the participant to withdraw, from their allocated treatment arm as over 80% of all transfusions are administered within 24 h of surgery, when the majority of patients will remain sedated and/or ventilated. If a patient wishes to withdraw for any reason, we will continue to analyse any data already collected, unless the patient expresses a wish for their samples and any associated data to be destroyed. On the basis of an ongoing trial measuring similar end points (the PASPORT Trial ISRCTN23557269), we predict that data may be incomplete for approximately 10% of patients.

#### Measures taken to avoid bias

All necessary steps will be taken to reduce the risk of bias.^44^ ^45^ The trial will be analysed on an intention-to-treat basis, that is, outcomes will be analysed according to the treatment allocation, irrespective of future management and events, and every effort will be made to include all randomised patients. Selection bias will be minimised by concealed randomised allocation. As the intervention utilises a large piece of bedside equipment, the autotransfusion device, it will be impossible to blind clinical staff and patients to the intervention. Detection bias will be minimised, however, by blinding of laboratory staff analysing cytokines, biomarkers and inflammatory processes. Specifically, urine and serum samples obtained exclusively for the trial will be identified only by a trial acronym, patient’ study ID, initials and date of birth, and the time at which the sample was taken, ensuring that laboratory staff performing analyses are blinded. Detection bias for the clinical outcomes will also be minimised by the use of objective outcome criteria; as defined in the Outcome section above. Staff recording components of the secondary outcome (any inflammatory injury, sepsis or death) at the time of discharge will be blinded to further minimise the risk of detection bias. Blood and other urine samples, that are also obtained as part of routine care will be analysed routinely in NHS laboratories by personnel who are unaware that the participant is in a trial. Decisions about discharge from ICU, HDU and from hospital will be made by clinical staff on the basis of existing institutional protocols. ICU/HDU transition will be defined as transition from level 3 (1:1 nursing ratio) to level 2 (1:2 nursing ratio). HDU/ward transition will be defined as time of arrival on the ward. In order to minimise attrition bias, we aim to include data for all randomised participants in the data analyses.

#### Adverse events

Serious and other adverse events are recorded and reported in accordance with the International Conference for Harmonisation of Good Clinical Practice (ICH GCP) guidelines and the Sponsor's (University of Leicester) Research Related Adverse Event Reporting Policy. University Hospitals of Coventry and Warwickshire Trust and Royal Victoria Hospital Blackpool will notify the trial team of all serious adverse events. Data on adverse events are collected from the time of surgery for the duration of the participant's postoperative hospital stay and for the 3-month follow-up period.

### Statistical analyses

The primary outcome, serum IL-8 levels, is continuously scaled, so the target differences can be specified as a ‘standardised differences’ (0.2=small, 0.5=moderate, 0.8=large). On the assumption that there will be a moderate correlation of 0.7 between preintervention and postintervention measures and between repeated postintervention measures, as observed in previous work,^46^ and on the basis that there will be one baseline and five postoperative measures, we estimate that a sample size of 150 patients will allow us to detect a small-to-moderate target difference between groups of 0.4, with 90% power and 5% significance (two-tailed). We propose to recruit 170 patients (85 per group) assuming an attrition rate of between 10% and 15% for incomplete sampling, patient death and withdrawal.

This sample size is not expected to detect significant differences in important clinical outcomes. In a previous trial that recruited patients at increased risk of transfusion we observed the composite end point of any sepsis, inflammatory organ injury or death in 55% of patients.^36^ If this is replicated in the REDWASH trial, our sample size will allow us to detect a 40% difference in with 80% power and 5% significance. To detect a more modest reduction in this composite end point (20%) would require at least 324 patients per group. Specific morbidities and other adverse events are too infrequent for the trial to be able to detect differences between groups. Frequencies of these adverse outcomes will be tabulated, in line with guidelines for reporting adverse events in trials.^45^

#### Plan of analysis

The primary analysis will take place when follow-up is complete for all patients and will be performed on an intention-to-treat basis. Means for continuous outcomes (transformed logarithmically if required) will be compared using analysis of variance or regression modelling, adjusting for baseline values where available. Findings will be reported as effect sizes with 95% CIs. Time to classification as fit for discharge, ICU and postoperative hospital stay will be analysed as time-to-event data using regression modelling for survival data. Frequencies of adverse clinical outcomes will be tabulated, in line with guidelines for reporting adverse events in trials.^45^ A mechanism substudy will consider links between markers of inflammation, cellular and platelet activation, and organ injury, in the first 60 participants from Glenfield Hospital (approximately 30 for each group).

#### Sensitivity analyses

Sensitivity analyses will include (1) per-protocol analyses and safety analyses will consider the likely effects of patient withdrawals and protocol non-compliance on the trial results; (2) consider the interaction between the intervention and the age of blood transfused, that is, those who receive only blood less than 14 days old versus those that receive any blood over 14 days old, on the basis that the proposed intervention is expected to prevent the risks attributed to prolonged blood storage.

#### Frequency of analysis

The primary analysis will take place when follow-up is complete for all recruited patients. Outcome data will be reported to the Data Monitoring and Safety Committee every 6 months, together with any additional analyses the committee request. In these reports, the data will be presented by group.

#### Economic issues/analysis

Health economic analysis will be undertaken by the Health Economics Research Centre of the University of Oxford. Established guidelines will be followed for the economic evaluation.^47^ The main outcome measure will be quality-adjusted life years (QALYs), estimated using the EuroQol EQ5D.^39^ This questionnaire instrument will be administered face-to-face to patients at baseline and at 6 weeks. At 3 months postrandomisation, this questionnaire instrument will be administered via post to patients. Respondents will be assigned valuations derived from published UK population tariffs^48^ and the mean number of QALYs per trial arm and incremental QALYs will be calculated. Patients will be followed up for 6 weeks after surgery, at a routine outpatient clinic appointment and again at 3 months postrandomisation via postal questionnaire.

Data will be collected from the trial centres on healthcare resource use for transfusion, inpatient days by ward type, any complications and subsequent treatments for complications. Resource use will be measured in naturally occurring units; for example, staff time will be measured in terms of length of times for treatments and unit costs will be derived from nationally published sources.^43^ Bespoke questionnaires will be used at 6 weeks and 3 months postrandomisation to obtain estimates of healthcare resources used since hospital discharge, for example, readmissions to hospital and further contact with health professionals such as general practitioner visits.

Any missing outcome and cost data will be dealt with using multiple imputation methods. Our analysis will calculate the average cost and outcome on a per patient basis and from this the incremental cost-effectiveness ratios for the different trial arms will be derived, producing an incremental cost per QALY, or incremental cost per complication avoided. Probabilistic sensitivity analysis will be used to assess the impact on results of variation around key parameters such as costs for treatments for complications which will also assist with generalising the results to other UK settings. Results will be expressed in terms of a cost-effectiveness acceptability curve, which indicates the likelihood that the results fall below a given cost-effectiveness ceiling.

## Trial management

The trial will be managed by the Cardiac Surgery Clinical Trials Team at the University of Leicester, supported by the Leicester Clinical Trials Unit (CTU), a UK Clinical Research Collaboration registered Clinical Trials Unit. The South West Leicestershire Research Ethics Committee approved the trial protocol on 15 May 2013 (REC Reference 12/EM/0475).

## Patient and public involvement

The Leicester Cardiac Surgery (LCS) Patient and Public Involvement (PPI) group brings together cardiac patients, some of whom have participated in clinical trials, and members of the public, many of whom have PPI clinical research experience in local and national organisations. LCS PPI group members actively participate in research activities. A consultation exercise with the entire PPI group has informed the study design and selection of clinical end points. Three members of the consultation subgroup have contributed to the authorship of this paper. The consultation subgroup has also contributed to the drafting of information leaflets for patients and their relatives in the trial. PPI group members are established within the research governance committees for the trial. PPI group members are also networked to local and national PPI groups and this is an additional resource that we have used to inform our recruitment processes. Group members are currently conducting a pilot evaluation of patient participation (enhanced patient visitor role) for patients in the REDWASH trial. A dissemination subgroup will coordinate local and national public dissemination activities.

## Dissemination

The data from the REDWASH study will be available for further ethically approved research studies. The findings will be disseminated by usual academic channels, that is, presentation at international meetings, as well as by peer-reviewed publications and through patient organisations and newsletters to patients, where available. As the study evaluates technology that is already ubiquitous in high-risk cardiac surgery, we do not predict that there will be commercially exploitable findings from this study.

## References

[1] Murphy GJ, Ascione R, Angelini GD Coronary artery bypass grafting on the beating heart: surgical revascularisation for the next decade. Eur Heart J 2004;25:2077–85. 10.1016/j.ehj.2004.09.022 15571822

[2] Mangano CM, Diamondstone LS, Ramsay JG Renal dysfunction after myocardial revascularization: risk factors, adverse outcomes, and hospital resource utilization. The Multicenter Study of Perioperative Ischemia Research Group. Ann Intern Med 1998;128:194–203. 10.7326/0003-4819-128-3-199802010-00005 9454527

[3] Dasta JF, Kane-Gill SL, Durtschi AJ Costs and outcomes of acute kidney injury (AKI) following cardiac surgery. Nephrol Dial Transplant 2008;23:1970–4. 10.1093/ndt/gfm908 18178605

[4] Rajakaruna C, Rogers CA, Angelini GD Risk factors for and economic implications of prolonged ventilation after cardiac surgery. J Thorac Cardiovasc Surg 2005;130:1270–7. 10.1016/j.jtcvs.2005.06.050 16256778

[5] Koch C, Li L, Figueroa P Transfusion and pulmonary morbidity after cardiac surgery. Ann Thorac Surg 2009;88:1410–18. 10.1016/j.athoracsur.2009.07.020 19853083

[6] Zacharias M, Mugawar M, Herbison GP Interventions for protecting renal function in the perioperative period. Cochrane Database Syst Rev 2013;(9):CD003590. 24027097 10.1002/14651858.CD003590.pub4 PMC7154582

[7] Patel NN, Rogers CA, Angelini GD Pharmacological therapies for the prevention of acute kidney injury following cardiac surgery: a systematic review. Heart Fail Rev 2011;16:553–67. 10.1007/s10741-011-9235-5 21400231

[8] Garwood S Renal insufficiency after cardiac surgery. Semin Cardiothorac Vasc Anesth 2004;8:227–41. 10.1177/108925320400800305 15375482

[9] Clark SC Lung injury after cardiopulmonary bypass. Perfusion 2006;21:225–8. 10.1191/0267659106pf872oa 16939116

[10] Bridgewater B, Keogh B Demonstrating quality: the Society of Cardiothoracic Surgeons of Great Britain and Ireland National Sixth Adult Cardiac Surgical Database Report 2008. Oxforshire, United Kingdom: Dendrite Clinical Systems.

[11] Murphy MF, Murphy GJ, Gill R 2011 Audit of Blood Transfusion in Adult Cardiac Surgery. Published by the National Comparative Audit of Blood Transfusion, 2013.

[12] Bennett-Guerrero E, Zhao Y, O'Brien SM Variation in use of blood transfusion in coronary artery bypass graft surgery. JAMA 2010;304:1568–75. 10.1001/jama.2010.1406 20940382

[13] Murphy GJ, Reeves BC, Rogers CA Increased mortality, postoperative morbidity, and cost after red blood cell transfusion in patients having cardiac surgery. Circulation 2007;116:2544–52. 10.1161/CIRCULATIONAHA.107.698977 17998460

[14] Koch CG, Li L, Duncan AI Morbidity and mortality risk associated with red blood cell and blood-component transfusion in isolated coronary artery bypass grafting. Crit Care Med 2006;34:1608–16. 10.1097/01.CCM.0000217920.48559.D8 16607235

[15] Patel NN, Avlonitis VS, Jones HE Indications for red cell transfusion in cardiac surgery: a systematic review and meta-analysis of randomised controlled trials and observational studies. Lancet Haematol 2015;2:e543–53. 10.1016/S2352-3026(15)00198-2 26686409

[16] Karkouti K, Wijeysundera DN, Yau TM The independent association of massive blood loss with mortality in cardiac surgery. Transfusion 2004;44:1453–62. 10.1111/j.1537-2995.2004.04144.x 15383018

[17] Goudie R, Sterne JAC, Verheyden V Risk scores to facilitate preoperative prediction of transfusion and large volume blood transfusion associated with adult cardiac surgery. Br J Anaesth 2015;114:757–66. 10.1093/bja/aeu483 25904607

[18] Tinmouth A, Fergusson D, Yee IC, ABLE Investigators, Canadian Critical Care Trials Group. Clinical consequences of red cell storage in the critically ill. Transfusion 2006;46:2014–27. 10.1111/j.1537-2995.2006.01026.x 17076859

[19] Patel NN, Lin H, Jones C Interactions of cardiopulmonary bypass and erythrocyte transfusion in the pathogenesis of pulmonary dysfunction in swine. Anesthesiology 2013;119:365–78. 10.1097/ALN.0b013e31829419d3 23619171

[20] Looney MR, Nguyen JX, Hu Y Platelet depletion and aspirin treatment protect mice in a two-event model of transfusion-related acute lung injury. J Clin Invest 2009;119:3450–61. 19809160 10.1172/JCI38432 PMC2769181

[21] Patel NN, Toth T, Jones C Reversal of anaemia with allogenic RBC transfusion prevents post cardiopulmonary bypass acute kidney injury in swine. Am J Physiol Renal Physiol 2011;301:F605–14. 10.1152/ajprenal.00145.2011 21653630 PMC3174544

[22] Hod EA, Zhang N, Sokol SA Transfusion of red blood cells after prolonged storage produces harmful effects that are mediated by iron and inflammation. Blood 2010;115:4284–92. 10.1182/blood-2009-10-245001 20299509 PMC2879099

[23] Verhoeven AJ, Hilarius PM, Dekkers DW Prolonged storage of red blood cells affects aminophospholipid translocase activity. Vox Sang 2006;91:244–51. 10.1111/j.1423-0410.2006.00822.x 16958837

[24] Simak J, Gelderman MP Cell membrane microparticles in blood and blood products: potentially pathogenic agents and diagnostic markers. Trans Med Rev 2006;20:1–26. 10.1016/j.tmrv.2005.08.001 16373184

[25] Mause SF, Weber C Microparticles: protagonists of a novel communication network for intercellular information exchange. Circ Res 2010;107:1047–57. 10.1161/CIRCRESAHA.110.226456 21030722

[26] Carless PA, Henry DA, Moxey AJ Cell salvage for minimising perioperative allogeneic blood transfusion. Cochrane Database Syst Rev 2010;(4):CD001888. 10.1002/14651858.CD001888.pub4 PMC4163967 20393932

[27] Swindell CG, Barker TA, McGuirk SP Washing of irradiated red blood cells prevents hyperkalaemia during cardiopulmonary bypass in neonates and infants undergoing surgery for complex congenital heart disease. Eur J Cardiothorac Surg 2007;31:659–64. 10.1016/j.ejcts.2007.01.014 17291775

[28] Cholette JM, Henrichs KF, Alfieris GM Washing red blood cells and platelets transfused in cardiac surgery reduces postoperative inflammation and number of transfusions: results of a prospective, randomized, controlled clinical trial. Pediatr Crit Care Med 2012;13:290–9. 10.1097/PCC.0b013e31822f173c 21926663 PMC3839819

[29] Bennett-Guerrero E, Kirby BS, Zhu H Randomized study of washing 40- to 42-day-stored red blood cells. Transfusion 2014;54:2544–52. 10.1111/trf.12660 24735194 PMC4194130

[30] Walpoth BH, Eggensperger N, Walpoth-Aslan BN Qualitative assessment of blood washing with the continuous autologous transfusion system (CATS). Int J Artif Organs 1997;20:234–9. 9195242

[31] Levey AS, Bosch JP, Lewis JB A more accurate method to estimate glomerular filtration rate from serum creatinine: a new prediction equation. Modification of Diet in Renal Disease Study Group. Ann Intern Med 1999;130:461–70. 10.7326/0003-4819-130-6-199903160-00002 10075613

[32] Kidney Disease: Improving Global Outcomes (KDIGO) Acute Kidney Injury Work Group. KDIGO clinical practice guideline for acute kidney injury. Kidney Int Suppl 2012;2:1–138. 10.1038/kisup.2012.1

[33] Hennein HA, Ebba H, Rodriguez JL Relationship of the proinflammatory cytokines to myocardial ischemia and dysfunction after uncomplicated coronary revascularization. J Thorac Cardiovasc Surg 1994;108:626–35. 7934095

[34] Cremer J, Martin M, Redl H Systemic inflammatory response syndrome after cardiac operations. Ann Thorac Surg 1996;61:1714–20. 10.1016/0003-4975(96)00055-0 8651772

[35] Murphy GJ, Patel NN, Curry N Blood management. In: Alston P, Myles PS, Ranucci M, eds. Oxford textbook of cardiothoracic anaesthesia. Oxford University Press, 2015:191–201.

[36] Murphy GJ, Pike K, Rogers CA TITRe2 Investigators. Liberal or restrictive transfusion after cardiac surgery. N Engl J Med 2015;372:997–1008. 10.1056/NEJMoa1403612 25760354

[37] Ranieri VM, Rubenfeld GD, Thompson BT, ARDS Definition Task Force. Acute respiratory distress syndrome: the Berlin definition. JAMA 2012;307:2526–33. 22797452 10.1001/jama.2012.5669

[38] Marshall JC, Cook DJ, Christou NV Multiple organ dysfunction score: a reliable descriptor of a complex clinical outcome. Crit Care Med 1995;23:1638–52. 10.1097/00003246-199510000-00007 7587228

[39] EuroQol Group. EuroQol—a new facility for the measurement of health-related quality of life. Health Policy 1990;16:119–208. 10.1016/0168-8510(90)90421-9 10109801

[40] Haase M, Bellomo R, Devarajan P Accuracy of neutrophil gelatinase associated lipocalin (NGAL) in diagnosis and prognosis in acute kidney injury: a systematic review and metaanalysis. Am J Kidney Dis 2009;54:1012–24. 10.1053/j.ajkd.2009.07.020 19850388

[41] Portilla D, Dent C, Sugaya T Liver fatty acid-binding protein as a biomarker of acute kidney injury after cardiac surgery. Kidney Int 2008;73:465–72. 10.1038/sj.ki.5002721 18094680

[42] Fergusson DA, Hébert PC, Mazer CD BART Investigators. A comparison of aprotinin and lysine analogues in high-risk cardiac surgery. N Engl J Med 2008;358:2319–31. 10.1056/NEJMoa0802395 18480196

[43] http://www.dh.gov.uk/health/2012/01/reference-costs-manua/

[44] Higgins JPT, Altman DG Chapter 8: assessing risk of bias in included studies. In: Higgins JPT, Green S, eds. Cochrane handbook for systematic reviews of interventions version 5.0.1 (updated September 2008). The Cochrane Collaboration, 2008.

[45] Moher D, Schulz KF, Altman DG The CONSORT statement: revised recommendations for improving the quality of reports of parallel-group randomised trials. Lancet 2001;357:1191–4. 10.1016/S0140-6736(00)04337-3 11323066

[46] Rogers CA, Pike K, Kounali D A randomised controlled trial of median sternotomy vs. anterolateral left thoracotomy on morbidity and healthcare resource use in patients having off-pump coronary artery bypass surgery (SteT). J Thorac Cardiovasc Surg 2013;146:306–16. 10.1016/j.jtcvs.2012.04.020 22944093

[47] Weinstein MC, Siegel JE, Gold MR Recommendations of the panel on cost-effectiveness in health and medicine. JAMA 1996;276:1253–8. 10.1001/jama.1996.03540150055031 8849754

[48] Kind P, Dolan P, Gudex C Variations in population health status: results from a United Kingdom national questionnaire survey. BMJ 1998;316:736–41. 10.1136/bmj.316.7133.736 9529408 PMC28477

